# Rapid RNA Exchange in Aqueous Two-Phase System and Coacervate Droplets

**DOI:** 10.1007/s11084-014-9355-8

**Published:** 2014-02-28

**Authors:** Tony Z. Jia, Christian Hentrich, Jack W. Szostak

**Affiliations:** 1 Howard Hughes Medical Institute, Department of Molecular Biology, and Center for Computational and Integrative Biology, Massachusetts General Hospital, 185 Cambridge Street, Boston, MA 02114 USA; 2 Department of Chemistry and Chemical Biology, Harvard University, 12 Oxford St., Cambridge, MA 02138 USA

**Keywords:** Prebiotic chemistry, Phase separation, Compartmentalization, Aqueous two-phase systems, Coacervates, Origin of life

## Abstract

Compartmentalization in a prebiotic setting is an important aspect of early cell formation and is crucial for the development of an artificial protocell system that effectively couples genotype and phenotype. Aqueous two-phase systems (ATPSs) and complex coacervates are phase separation phenomena that lead to the selective partitioning of biomolecules and have recently been proposed as membrane-free protocell models. We show in this study through fluorescence recovery after photobleaching (FRAP) microscopy that despite the ability of such systems to effectively concentrate RNA, there is a high rate of RNA exchange between phases in dextran/polyethylene glycol ATPS and ATP/poly-L-lysine coacervate droplets. In contrast to fatty acid vesicles, these systems would not allow effective segregation and consequent evolution of RNA, thus rendering these systems ineffective as model protocells.

**Electronic supplementary material:**

The online version of this article (doi:10.1007/s11084-014-9355-8) contains supplementary material, which is available to authorized users.

## Introduction

The RNA world hypothesis provides a conceptual framework for the early development of life on earth in which RNA functions both as a molecule capable of propagating genetic information and as a catalyst. The capacity of RNA to transmit genetic information is exemplified by the RNA viruses, which can have genomes up to 30 kb in length consisting entirely of RNA (Lai and Cavanagh 1997). Ribozymes generated by in vitro directed RNA sequence evolution (Ellington and Szostak 1990; Tuerk and Gold 1990) and natural ribozymes such as self-splicing introns (Cech et al. 1981; Kruger et al. 1982) are important examples of catalytic RNAs that serve as paradigms for the catalytic role of RNA in a prebiotic world. RNA molecules with RNA polymerase activity have been evolved in the laboratory (Johnston et al. 2001; Attwater et al. 2013), and a pair of RNA ligase ribozymes have been shown to cross-replicate each other by ligation in an exponential manner (Lincoln and Joyce 2009). Although RNA-catalyzed RNA replication is likely to have been important for primitive cells in the RNA world, it is also possible that non-enzymatic RNA replication may have played an important role in the transition from prebiotic chemistry to the emergence of the first cells. Since at this stage neither RNA-catalyzed nor purely chemically driven RNA replication have been demonstrated, it is reasonable to consider routes to the assembly of protocells based on either mode of RNA replication. In either case, replicating RNAs must be compartmentalized to allow for the evolution of functional RNAs that confer a selective advantage to the protocell within which they reside.

While there has been great progress in understanding prebiotically plausible vesicle assembly and replication pathways (Budin and Szostak 2010; Chen and Walde 2010), combining both encapsulation and replication into a functional model protocell presents additional challenges. Compartmentalization of genomic RNA molecules without (or with only rare) exchange between protocells is essential for any protocell model as it would allow RNA sequences with desirable properties, such as catalytic ribozymes, to be segregated from other RNAs and to selectively replicate and evolve over time (Szostak et al. 2001; Szabo et al. 2002). Phospholipids are the major building blocks in modern cell membranes, however phospholipid membranes are largely impermeable to charged molecules (Chen and Walde 2010) and are therefore problematic as the basis of protocell compartmentalization. However, membranes composed of fatty acids and related single chain amphiphiles are permeable to small polar and even charged molecules, and have additional properties that are favorable for protocell growth and division (Budin and Szostak 2011). Nevertheless, the simplicity of membrane free protocell models is intriguing and makes such systems worth further exploration.

Droplets formed by phase separation in an aqueous environment, such as aqueous two-phase systems (ATPS) and charge-complex coacervates, have been proposed as model protocells (Oparin 1953; Fox 1976; Liebl et al. 1984; Koga et al. 2011; Keating 2012; Mann 2012, 2013). Both ATPSs (Albertsson 1971; Walter et al. 1985; Zaslavsky 1995) and coacervates (Dufrenoy and Reed 1946; Oparin et al. 1961) have long been known to lead to the partitioning of specific molecules into different phases in an overall aqueous environment. In biotechnological applications, ATPSs composed of dextran and polyethylene glycol (PEG) are commonly used to partition whole bacterial cells (Stendahl et al. 1977), cellular organelles (Albertsson 1958), and macromolecules (Hatti-kaul 2001); RNA, for example, partitions into the more polar dextran-rich phase (Zaslavsky 1992). Some properties of ATPSs and coacervates could have been advantageous in the development of early cells. Their ability to concentrate primitive reactants and catalysts, such as ribozymes, could increase reaction rates without requiring a lipid-based boundary (Strulson et al. 2012). Both ATPSs and coacervates also function as compartments in vitro (Williams et al. 2012; Strulson et al. 2012) and in the case of a dextran/PEG ATPS, within a phospholipid vesicle (Helfrich et al. 2002; Long et al. 2005). Coacervate droplets are particularly attractive due to the simplicity of their components, e.g. mononucleotides and small polypeptides, both of which could have been produced in a prebiotic environment (Leman et al. 2004; Powner et al. 2009).

Because of the membrane-independent nature of ATPS and coacervate models, it is unclear whether these systems are able to compartmentalize genetic molecules such as RNA with minimal exchange between droplets. We have therefore studied the ability of ATPS and coacervate droplets to retain RNA oligonucleotides 15 and 50 nucleotides in length, and thereby gauge their effectiveness as membrane-free protocell model systems.

## Results

### Properties of ATPS and Coacervate Systems

A 16 % dextran/10 % PEG (initial *w/v*) ATPS was prepared, yielding roughly equal volumes of the dextran-rich and PEG-rich phases (Fig. S1a). When the ATPS was mixed by vortexing, a turbid suspension consisting of small, dispersed dextran-rich droplets in the bulk PEG-rich phase and PEG-rich droplets in the bulk dextran-rich phase formed. After several minutes the droplets began to coalesce and the system separated into two clear phases (Fig. S1b), with the dextran-rich phase at the bottom due to its greater density. Whether the system was in a dispersed or a coalesced state, we observed a rapid 8-fold enrichment of a fluorescently labeled RNA 15-mer into the dextran-rich phase; the fluorescent dye did not have a strong effect on partitioning (Table S1).

We also investigated partitioning of RNA in ATPSs made using PEG and ionic derivatives of dextran, including cationic diethylaminoethyl dextran (DEAE-dextran) and anionic dextran-sulfate (Fig. S2). As expected, both of the PEG/dextran derivative systems lead to a greater degree of partitioning of RNA (Table S1). In a 25 % DEAE-dextran/25 % PEG (*w/v*) system (yielding ≈ 55 % DEAE-dextran-rich phase by volume), RNA partitioned strongly into the DEAE-dextran-rich phase due to the positive charge of the DEAE-dextran and the more polar nature of that phase; the degree of partitioning was so great that the RNA concentration in the PEG-rich phase was below our detection limit (Table S1). Conversely, in a 16 % dextran-sulfate/10 % PEG (*w/v*) system (≈60 % dextran-sulfate-rich phase by volume), RNA partitioned strongly into the PEG-rich phase, presumably due to charge repulsion from the anionic dextran-sulfate. Droplets in the DEAE-dextran/PEG system coalesced more slowly than droplets in the dextran/PEG or dextran-sulfate/PEG system (Fig. S3), most likely due to the high viscosity of DEAE-dextran. In all systems, renewed vortexing or mixing led to the reformation of the turbid state consisting of small, dispersed droplets.

We also prepared coacervates consisting of complexes of anionic ATP and cationic poly-L-lysine (pLys). Upon visual inspection, the ATP/pLys system (30 mM ATP, 2 % pLys) appeared similar to the ATPSs as two phases formed under specific concentration conditions (Fig. S4a). Following coalescence, the lower, more dense phase was highly enriched in ATP/pLys complexes formed by the charge balancing of these species (Fig. S4b). In order for coacervates to form, a number of parameters must fall within defined limits, including the ATP and pLys concentrations (Koga et al. 2011), salt concentrations (Fig. S5), nucleotides (Table S2), and the molecular weight of the pLys (Table S2). RNA oligomers partitioned strongly into the complex-enriched phase to a degree that was comparable to that of the DEAE-dextran/PEG system (Table S1).

### RNA Retention in ATPS and Coacervate Droplets

**Fig. 1:**
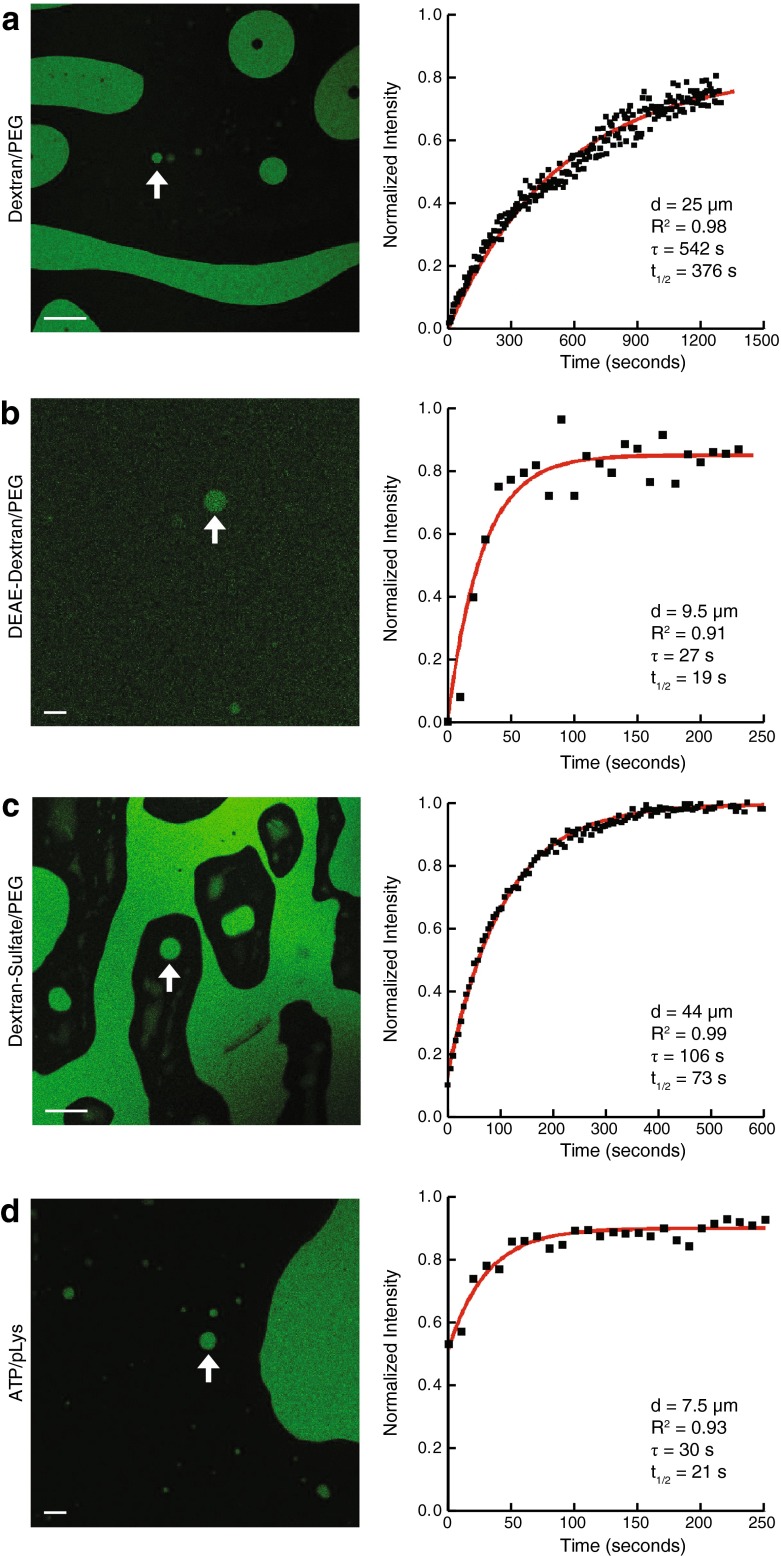
Rapid exchange of RNA oligomers between ATPS and coacervate droplets and the surrounding bulk phase. Representative confocal fluorescence images showing RNA enriched droplets (*green*) are shown at left. Normalized fluorescence recovery after photobleaching (FRAP) recovery curves are shown at right. All samples contained 5 μM 5′-6-FAM-labeled RNA 15-mer (5′-CCAGUCAGUCUACGC-3′) in: **(a)** 16 % *w/v* dextran 9-11 kDa/10 % *w/v* PEG 8 kDa in 50 mM Tris-Cl pH 8 and 100 mM NaCl (indicated droplet 25 μm diameter), **(b)** 25 % *w/v* DEAE-dextran >500 kDa/25 % *w/v* PEG 8 kDa in 100 mM Tris-Cl pH 8 with the GODCAT (glucose oxidase/catalase) system (*Methods*) (indicated droplet 9.5 μm diameter), **(c)** 16 % *w/v* dextran-sulfate 9-20 kDa/10 % *w/v* PEG 8 kDa in 50 mM Tris-Cl pH 8 and 100 mM NaCl (indicated droplet 44 μm diameter), **(d)** 30 mM ATP/2 % *w/v* pLys 4-15 kDa in 100 mM Tris-Cl pH 8 with the GODCAT system (*Methods*) (indicated droplet 7.5 μm diameter). See Movies S1-S4 for respective FRAP movies. Each curve was normalized to the intensities of a non-bleached droplet and the background within the same frame, to correct for photobleaching during sampling, as well as to its initial intensity, to account for variable photobleaching before the recovery step across runs (Supplementary Information). Data were fit to a single exponential to determine time constants (τ) and half-lives (t_1/2_) for fluorescence recovery (Supplementary Information). Further details and data in Table S3. Scale bars for **(a)** and **(c)** are 100 μm; scale bars for **(b)** and **(d)** are 10 μm. See Movies S1-S4 for full movies of photobleaching and recovery for each of the indicated droplets in **(a)**-**(d)**, respectively

We sought to determine the ability of ATPS and coacervate droplets to retain RNA in a manner similar to fatty acid based vesicles by preparing droplets into which a fluorescently labeled RNA 15-mer oligonucleotide had partitioned. We then used fluorescence recovery after photobleaching (FRAP) microscopy to analyze the rates at which the RNA moved from the bulk phase into photo-bleached droplets. At steady state, this would be equivalent to the rate at which RNA diffused out of droplets into the bulk phase (and then into other droplets). We acquired and analyzed fluorescence recovery data for fluorescently labeled RNA in droplets from four systems (Table S3): 16 % dextran/10 % PEG (Fig. 1a, Movie S1), 25 % DEAE-dextran/25 % PEG (Fig. 1b, Movie S2), 16 % dextran-sulfate/10 % PEG (Fig. 1c, Movie S3), and 30 mM ATP/2 % pLys (Fig. 1d, Movie S4) (all percentages *w/v*). The sizes of droplets ranged from 1 to 5 μm in diameter (Fig. S6), similar in size to proposed fatty acid vesicle based protocell model systems (Adamala and Szostak 2013a), up to 50–75 μm in diameter (Fig. 1c), similar in size to giant unilamellar vesicles (Dimova et al. 2006).

In dextran-rich and DEAE-dextran-rich droplets (in their respective ATPSs) between 5 μm and 10 μm in diameter, the fluorescence recovery half-life (t_1/2_) of the fluorescently labeled RNA oligonucleotides was 8–20 s (Table S3). In the dextran/PEG system, larger dextran-rich droplets (20 μm and 25 μm in diameter) (Fig. S7) recovered fluorescence significantly more slowly than the other dextran-rich droplets measured, possibly due to their larger size and/or their greater distance from other droplets. The fluorescence of RNA-enriched PEG-rich droplets in the dextran-sulfate/PEG ATPS, despite being the largest droplets sampled in all systems, recovered more quickly than large droplets in the dextran/PEG system (Table S3).

The RNA-enriched ATP/pLys droplets also recovered fluorescence quickly after photobleaching. The rate of exchange of RNA between droplets and their surrounding bulk phase was similar to that seen in dextran and DEAE-dextran droplets of comparable size (Table S3). After photobleaching, the fluorescence recovery t_1/2_ was 5–21 s for the ATP/pLys droplets measured (3–9 μm in diameter) (Table S3).

To test the influence of length on RNA retention within droplets, we measured the fluorescence recovery t_1/2_ after photobleaching of droplets of the dextran/PEG ATPS and the ATP/pLys system containing a fluorescently labeled RNA 50-mer. For the droplets measured in both of these systems, the fluorescence recovery t_1/2_ was 11–76 s (4–11 μm in diameter) (Table S4). Compared to similar-sized droplets in their respective systems containing the RNA 15-mer (Table S3), droplets containing the longer RNA resulted in a modest increase of the fluorescence recovery t_1/2_ by a factor of roughly 3.

**Fig. 2:**
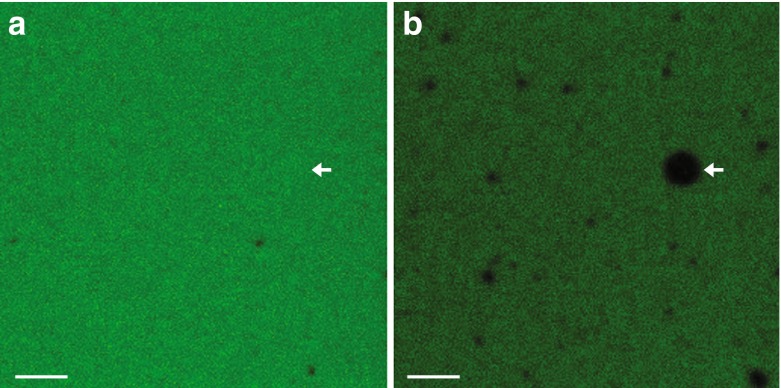
Oleic acid vesicles do not exchange RNA with the surrounding fluid. Representative confocal microscope images of a sample **(a)** before photobleaching and **(b)** 590 s after photobleaching of the indicated non-gel-filtered oleic acid vesicle in 200 mM Bicine-NaOH pH 8.5 containing 5′-6-FAM labeled RNA 15-mer (5′-CCAGUCAGUCUACGC-3′) at room temperature (*Methods*). The vesicle samples were not gel filtered in order to maintain a high RNA concentration outside of the vesicles in order to simulate conditions similar to the ATPS and coacervate systems. After the entire window was photobleached, fluorescence outside of the vesicles recovered due to rapid RNA diffusion, but fluorescence inside vesicles did not recover due to lack of transport of RNA across the membrane. Scale bars, 10 μm. See Movie S5 for full movie of photobleaching and recovery

To compare the time scale of RNA retention between phase-separated droplet systems and fatty acid vesicles, we prepared oleic acid vesicles, similar in size to the droplets studied above, that contained the fluorescently labeled RNA 15-mer. For the vesicle experiments, a high concentration of fluorescently labeled RNA was present outside of the vesicles as well. Ten minutes after photobleaching a sample, the external solution had fully recovered in fluorescence intensity due to the diffusion of RNA from adjacent non-bleached sample regions. However, the vesicles did not regain any detectable internal fluorescence intensity (Fig. 2, Movie S5). As expected, fatty acid vesicles, despite being more permeable to charged species than phospholipid vesicles, did not exhibit measurable permeability for RNA oligomers. The rate of RNA exchange across a fatty acid vesicle membrane was several orders of magnitude slower than the rate of RNA exchange across the boundaries of ATPS or coacervate droplets. This observation clearly demonstrates that although dextran/PEG ATPS and ATP/pLys coacervate systems are able to concentrate RNA molecules through partitioning into droplets, their ability to compartmentalize these oligomers over time scales longer than seconds was insignificant compared to that of fatty acid vesicles.

**Fig. 3:**
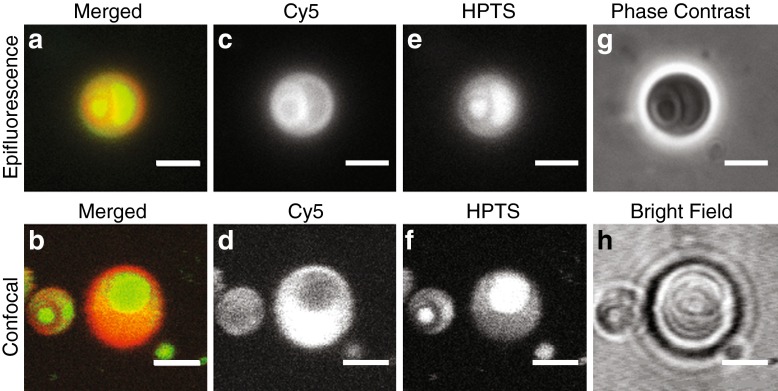
Formation of a dextran-PEG ATPS inside oleate vesicles. **(a)** and **(b)**: Merged images of Cy5-RNA fluorescence (*red*, Dextran-rich phase) and 8-hydroxypyrene-1,3,6-trisulfonate (HPTS) fluorescence (*green*, PEG-rich phase). **(c)** and **(d)**: the individual Cy5-RNA fluorescence channels for **(a)** and **(b)**, respectively. **(e)** and **(f)**: the HPTS fluorescence channels for **(a)** and **(b)**, respectively. **(g)** and **(h)**: Corresponding phase contrast (*top*) and bright field images (*bottom*). Images in the top row were acquired sequentially using an epifluorescence microscope; images in the bottom row were acquired simultaneously using confocal microscopy. Cy5-labeled RNA partitioned strongly into the dextran-rich phase, and HPTS partitioned into the PEG-rich phase. The dextran-rich (*red*) and the PEG-rich (*green*) phases could separate spontaneously within an oleic acid vesicle. See Methods for details. Scale bars, 3 μm

We then asked whether combining a dextran/PEG ATPS or an ATP/pLys coacervate system with current vesicle systems would allow RNA partitioning within a model protocell. Previous work has shown that it is possible to form phospholipid vesicles that contain dextran/PEG ATPSs (Helfrich et al. 2002; Long et al. 2005; Dominak et al. 2010), and that these systems are able to partition RNA to sub-regions within a vesicle. We were able to encapsulate a dextran/PEG ATPS inside oleic acid vesicles (Fig. 3). As expected, the fluorescently labeled RNA 15-mer partitioned into the dextran-rich phase inside oleate vesicles, providing an RNA-rich compartment within these vesicles. However, the ATP/pLys system used in this study was not compatible with fatty acids. Attempts to produce fatty acid vesicles containing the ATP/pLys system resulted in quantitative precipitation of the fatty acids, most likely due to the charge interactions between the cationic lysine side chain and anionic fatty acid molecules.

## Discussion

For the ATPS and coacervate droplets studied, exchange of RNA across the droplet boundary occurred orders of magnitude more rapidly than across the membrane of fatty acid vesicles. Although our FRAP measurements report only on the entry of RNA oligomers into ATPS or coacervate droplets, at steady state, the rate of efflux of RNA from droplets must equal the rate of influx. Our data therefore imply that RNA molecules do not remain localized within any droplet for longer than a period of seconds, and rapidly exchange between droplets via the surrounding bulk phase. Although a larger RNA such as a ribozyme would diffuse more slowly in solution due to its greater mass, our data indicates that longer RNAs will not reside in a droplet for a significantly longer time before diffusing out of the droplet. Fast RNA exchange coupled with the observed rapid coalescence of droplets suggests that ATPS and coacervate droplets would not confer the stable compartmentalization necessary for multiple generations of RNA selection and replication to occur, which would need to be on the order of many hours, if not days (Deck et al. 2011; Adamala and Szostak 2013b). If a given RNA molecule only resides in a particular droplet for a short period of time before exchanging into a different droplet, the products of any functional activity of that RNA (such as the catalytic production of a useful metabolite) would be spread across many droplets, and furthermore would not be heritable. In essence, the rapid exchange of RNA molecules between droplets is equivalent to a lack of compartmentalization in a time-averaged sense. Darwinian evolution requires compartmentalization so that mutations that improve function can lead to a selective advantage for the mutant genomic molecule. As the capacity for Darwinian evolution is a basic requirement for any protocell model, it is clear that unmodified ATPS and coacervate droplets are unsuitable protocell models.

To decrease the rate of RNA exchange between droplets, it may be productive to consider systems in which RNA molecules could covalently attach to a matrix or to particles that would stay localized within a droplet. Many RNA affinity purification techniques rely on covalent attachments to a matrix such as sepharose (Allerson et al. 2003) or agarose beads (Caputi et al. 1999) and such a system could serve to slow RNA exchange. The coacervate system we studied was composed of a simple polypeptide (pLys) and a simple mononucleotide (ATP). RNA-protein (Lee et al. 1977; Drygin 1998; Baskerville and Bartel 2002) or RNA-nucleotide (Flügel and Wells 1972; Flügel et al. 1973) covalent interactions produced by photo-crosslinking could be good starting points to develop a system in which RNA becomes covalently linked to a matrix within coacervate droplets in a prebiotically plausible manner. Alternatively, utilizing a poly(Arg) matrix could result in tightly bound RNA that is unable to diffuse away from the droplet due to the electrostatic interactions between the arginine side-chains and the RNA backbone (Knight and Landweber 1998). Other immobilization techniques that take advantage of the ability of RNA to form base-pairs could also serve to slow RNA exchange.

Although dextran/PEG ATPS and ATP/pLys coacervate systems do not provide suitably stable compartmentalization of reactants for long periods of time, such systems do enable transient localization and concentration of RNA molecules. Focusing on the potential usefulness of these systems for sub-compartmentalization within protocells may be a productive direction for future research (Hyman and Brangwynne 2011). Fatty acid and phospholipid vesicle systems compatible with dextran/PEG ATPSs have been developed (Helfrich et al. 2002; Long et al. 2005; Dominak et al. 2010; this study), and it may be possible to develop similar vesicle systems that are compatible with the ATP/pLys coacervate system. This might be achieved by using net-neutral zwitterionic phospholipids or non-ionic amphiphiles as membrane forming molecules, as they would not interact strongly with the coacervate components, thus avoiding precipitation. Such a system would be similar to cellular organelle-based compartmentalization. In a prebiotic setting, a lipid-based membrane could encapsulate all components, and selective chemical partitioning into the two phases could provide an early protocell with the ability to partition compounds internally and accelerate reactions within the protocell, including for example the assembly of RNA complexes and ribozyme catalysis (Strulson et al. 2012). Thus, understanding how ATPSs and coacervates interact and combine with fatty acid and phospholipid vesicles may lead to a greater understanding of the possibilities for the development of early cells in an RNA world.

## Methods

### Chemicals

Tris(hydroxymethyl) aminomethane (Tris), sodium chloride, magnesium chloride hexahydrate, D-(+)-glucose, 2-mercaptoethanol, adenosine 5′-triphosphate (ATP) disodium salt hydrate, adenosine 5′-diphosphate (ADP) disodium salt, adenosine 5′-monophosphate (AMP) disodium salt, guanosine 5′-triphosphate (GTP) sodium salt hydrate, guanosine 5′-diphosphate (GDP) sodium salt, guanosine 5′-monophosphate (GMP) disodium salt hydrate, uridine 5′-triphosphate (UTP) trisodium salt hydrate, 8-hydroxypyrene-1,3,6-trisulfonic acid (HPTS) trisodium salt, enzyme catalase from bovine liver, polyethylene glycol (PEG) 8 kDa, dextran 9–11 kDa from *Leuconostoc mesenteroides*, dextran sulfate sodium salt 9–20 kDa from *Leuconostoc* spp., diethylaminoethyl-dextran (DEAE-dextran) hydrochloride >500 kDa, poly-L-lysine (pLys) hydrobromide 1–5 kDa, poly-L-lysine hydrobromide 4–15 kDa, poly-L-lysine hydrobromide 15–30 kDa, and Sepharose 4B (45–165 μm bead diameter) beads were purchased from Sigma-Aldrich Corporation (St. Louis, MO).

RNA oligonucleotides [(5′-CCAGUCAGUCUACGC-3′) both unlabeled and 6-carboxyfluorescein (6-FAM) 5′-labeled, 6-FAM 5′-labeled (5′- CAUCUAGUUACCUCUAGGAUCUCAUGAUGCCUGAAGCGUAGACUGACUGG-3′), and 5′-Cy5-labeled (5′-GCGUAGACUGACUGG-3′)] were purchased from Integrated DNA Technologies (Coralville, IA) and used without further purification. dNTPs and cytidine 5′-triphosphate (CTP) sodium salt were purchased from GE Healthcare (Little Chalfont, United Kingdom). Oleic acid was purchased from Nu-Chek Prep, Inc. (Elysian, MN). rNTPs and glass microscope slides (25 mm × 75 mm, 1 mm thick) were purchased from VWR International (Radnor, PA). Glucose oxidase from *Aspergillus* was purchased from Serva Electrophoresis (Heidelberg, Germany). Glass cover slips (18 × 18 mm No. 1) were purchased from Thermo Fisher Scientific (Waltham, MA). All solutions were produced in nuclease-free water from BioExpress (Kaysville, UT).

### Preparation of ATPS and Coacervate Samples

A 16 % *w/v* dextran 9–11 kDa and 10 % *w/v* PEG 8 kDa solution was prepared by dissolving the solid components in a solution of 50 mM Tris-Cl pH 8 and 100 mM NaCl (Strulson et al. 2012) with vigorous vortexing for a few minutes. The 16 % *w/v* dextran-sulfate sodium salt 9–20 kDa and 10 % *w/v* PEG 8 kDa was prepared by dissolving the solid components in a solution of 50 mM Tris-Cl pH 8 and 100 mM NaCl with moderate vortexing for several seconds. The 25 % *w/v* DEAE-dextran hydrochloride >500 kDa and 25 % *w/v* PEG 8 kDa was prepared by dissolving the solid components in a solution of 100 mM Tris-Cl pH 8 with vigorous vortexing and heating to 65 ^o^C for several minutes. 30 mM ATP - 2 % *w/v* pLys (either 1–5 kDa, 4–15 kDa, or 15–30 kDa as indicated) was prepared by mixing respective stock solutions (200 mM ATP and 10 % or 50 % *w/v* pLys both in 100 mM Tris-Cl pH 8) and diluting with 100 mM Tris-Cl pH 8. All samples were prepared in 1.5 mL eppendorf tubes at room temperature. Due to the viscosity of the DEAE-dextran/PEG sample, pipet tips that were cut roughly 1 cm from the tip were used for that sample. To each sample, 5′-6-FAM-labeled RNA (5′- CCAGUCAGUCUACGC-3′ or 5′-CAUCUAGUUACCUCUAGGAUCUCAUGAUGCCUGAAGCGUAGACUGACUGG-3′) from a 100 μM stock solution in nuclease-free water was added to a final concentration of 5 μM RNA. Each solution was vortexed for 30 s. For applications that required the two phases to be separated, the sample tube was centrifuged for 15 min at 14,000 rpm. Each phase was then pipetted into separate tubes. Transmittance measurements were performed using a GE Healthcare (formerly Amersham) Ultrospec 3,100 pro UV-Visible spectrometer (Little Chalfont, United Kingdom). RNA phase-specific partitioning measurements were performed using a Thermo Fisher Scientific (Waltham, MA) Nanodrop 2000c Spectrophotometer. For confocal microscopy, DEAE-dextran/PEG and ATP/pLys samples also contained the GODCAT system (Glucose Oxidase-Catalase) to reduce photobleaching (Hentrich and Surrey 2010), and included 2 % *w/v* D-(+)-glucose, 0.5 mg/mL catalase, 1 mg/mL glucose oxidase, and 143 mM 2-mercaptoethanol. A 6–8 μL droplet (6 μL for dextran/PEG, dextran-sulfate/PEG samples, and ATP/pLys samples, and 8 μL for DEAE-dextran/PEG samples) for each sample was applied to each glass slide (25x75mm) and a cover slip (18x18mm, No. 1) was applied. The slide was allowed to sit at room temperature until the droplet applied was completely spread across the entire cover slip area, and then the cover slip was sealed using Valap (1:1:1 vaseline, lanolin, paraffin wax) to avoid evaporation. Samples were covered with aluminum foil to reduce photobleaching by stray light until imaging.

### Preparation of Oleic Acid Vesicle Samples

~10 mM oleic acid vesicles containing 5′-6-FAM-labeled RNA (5′-CCAGUCAGUCUACGC-3′) were prepared by mixing 1.6 μL pure oleic acid (3.17 M) with 50 μL of 10 μM RNA in 500 μL 180 mM bicine buffer adjusted to pH 8.5 with NaOH, followed by vortexing for 30 s. The sample was covered with foil and allowed to gently tumble overnight. A 3 μL droplet was applied to a glass slide as above for microscopy. The glass slide was then allowed to sit (cover slip down) at room temperature for 30 min to allow larger vesicles to rest on the surface of the cover slip.

### Preparation of a Dextran/PEG ATPS Inside Oleic Acid Vesicles

To 840 μL of 5.95 % PEG 8 kDa, 10.7 % Dextran 10 kDa, 200 mM bicine pH 8.5 (adjusted with NaOH), 0.5 μL 200 mM HPTS (8-hydroxypyrene-1,3,6-trisulfonate, stock in H_2_O, 0.12 mM final concentration) and 10 μL of 100 μM 5′-Cy5-labeled RNA (5′-GCGUAGACUGACUGG-3′ in H_2_O, 1.2 μM final concentration) were added. The solution was vigorously vortexed and visually inspected to verify that it contained only one phase. Subsequently, 3 μL of oleic acid were added to the solution and after another vigorous vortexing, the solution was tumbled over night on a rotating wheel (6 rpm) to allow vesicle formation. The next day, the vesicles were purified from unencapsulated dye and RNA using a short 1 cm Sepharose 4B gel filtration column and 1 mM oleic acid in 200 mM bicine (adjusted to pH 8.5 with NaOH) as a running buffer. 6 μL of gel-filtered vesicles were spread out (to around 1 cm^2^) on a 25x75 mm microscope slide and the droplet was allowed to evaporate for 6 min at room temperature. Then an 18x18mm coverslip was placed onto the droplet and the slide was sealed using Valap. Alternatively, a 3 μL droplet was placed on a slide and a coverslip was placed immediately on top of it. In this case, the coverslip was not sealed, but only fixed in the corners with Valap, and evaporation was allowed to occur through the edges over several hours. Slides were observed either with a confocal microscope (see below) or with a Nikon (Tokyo, Japan) TE2000 inverted fluorescence microscope with a 100× oil objective.

### Fluorescence Recovery After Photobleaching (FRAP) by Confocal Microscopy

Each sample was imaged using a confocal microscope at 488 nm (pinhole 1 AU). Confocal microscopy was performed using a Leica (Solms, Germany) SP5 AOBS Scanning Laser Confocal Microscope (63×, 1.4-0.6 N. A. Plan-Apochromat oil immersion objective or 20×, 0.7 N. A. air objective), a Carl Zeiss (Oberkochen, Germany) LSM 510 Laser Scanning Microscope (63×, 1.4 N. A. Plan-Apochromat oil immersion objective), or a Nikon (Tokyo, Japan) A1R Confocal Microscope (60×, 1.49 N. A. Apochromat TIRF oil immersion objective). After selection of the droplet to be analyzed, a time zero image was acquired, and then a circular or square region was photobleached at high power using an Argon laser at 488 nm (or a solid state laser for the Nikon system). Each photobleaching region was chosen to be as small as possible while still containing a single, whole droplet to minimize collateral photobleaching of neighboring droplets. The fluorescence intensity (either 493 nm to 543 nm on the Leica system, 505 nm to 530 nm on the Zeiss system, or 500 nm to 550 nm on the Nikon system) was then measured over time to track the fluorescence recovery of 5′-6-FAM-labeled RNA molecules within the droplet of interest.

### Image and Data Analysis

Curve fitting of the fluorescence recovery after photobleaching (FRAP) intensities was carried out by first obtaining intensities across all time points of a specific droplet. These intensities were normalized to the intensities of a non-bleached droplet and the background within the same frame, to correct for nonspecific photobleaching during sampling. The intensities were then normalized to the initial intensity of the droplet analyzed, to account for variable photobleaching before the recovery step across runs (Phair et al. 2004). Curves were then fit to a single exponential recovery function. See Supplemental Information for detailed explanation of image analysis and curve fitting. All imaging visualization, analysis, calculations, and production of movies were performed using FIJI (Fiji is Just ImageJ). All curve fitting was performed using MATLAB (Natick, MA). All figures were produced using Adobe Illustrator (San Jose, CA).

## Electronic supplementary material

Below is the link to the electronic supplementary material.

Movie S1 (AVI 7949 kb)

Movie S2 (AVI 3858 kb)

Movie S3 (AVI 30671 kb)

Movie S4 (AVI 711 kb)

Movie S5 (AVI 1389 kb)

ESM 6 (PDF 3.00 mb)
