# Supplementary material for: Rapid RNA Exchange in Aqueous Two-Phase System and Coacervate Droplets
Source: Orig Life Evol Biosph. 2014 Feb 28;44(1):1–12. doi: 10.1007/s11084-014-9355-8 (PMC4141154; doi:10.1007/s11084-014-9355-8)
Supplement: Supplementary file 6 — (PDF 3.00 mb) [file 11084_2014_9355_MOESM6_ESM.pdf]

Supplementary Information

Rapid RNA Exchange in Aqueous Two-Phase System and Coacervate Droplets

Tony Z. Jia[1,2], Christian Hentrich[1], Jack W. Szostak[1,2*]

[1]Howard Hughes Medical Institute, Department of Molecular Biology, and Center for Computational and Integrative Biology, Massachusetts General Hospital, 185 Cambridge Street, Boston, Massachusetts, 02114

[2]Department of Chemistry and Chemical Biology, Harvard University, 12 Oxford St., Cambridge, Massachusetts, 02138

***Corresponding Author:**

Jack W. Szostak

Howard Hughes Medical Institute, Department of Molecular Biology, and Center for Computational and Integrative Biology

Massachusetts General Hospital

185 Cambridge Street, Massachusetts, 02114

Tel: 617-726-5981

Fax: 617-726-6893

Email: szostak@molbio.mgh.harvard.edu

Image Analysis and Curve Fitting

We utilized the following procedure to calculate the normalized fluorescence intensity of the sample at each time point, similar to the one used by Phair, et al. in 2004. Three values were obtained at all times t: **S(t)**, the average intensity within an arbitrarily defined region of interest entirely within the droplet analyzed; **B(t)**, the average intensity of an arbitrarily defined region of interest containing no droplets; and **C(t)**, the average intensity within an arbitrarily defined region entirely within a different droplet or large reservoir that exhibited no effects of the photobleaching step. The normalized intensity was then determined to be:

$$\frac{\left(\frac{S(t) - B(t)}{C(t) - B(t)}\right)}{\left(\frac{S(0) - B(0)}{C(0) - B(0)}\right)}$$

The normalized intensities were then fit to a single exponential recovery function:

$$y = A - Ae^{\frac{t}{\tau}} + C$$

y is the normalized fluorescence intensity, τ is the fluorescence recovery time constant, and A and C are constants.

The intensities for the droplet for all DEAE-dextran/PEG samples were normalized as follows: the normalized intensity (as shown above) in the first post-bleach frame was set to zero and the normalized intensity in the pre-bleach frame was set to one. This is due to the fact that photobleaching an area results in a lower raw intensity within that area compared to the rest of the background in the frame (Fig. 1b, Movie S2). This procedure does not change the fluorescence recovery time constant τ, only the constants A and C.

References

Phair RD, Gorski SA, Misteli T (2004) Measurement of dynamic protein binding to chromatin in vivo, using photobleaching microscopy. *Methods Enzymol* 375:393–414.

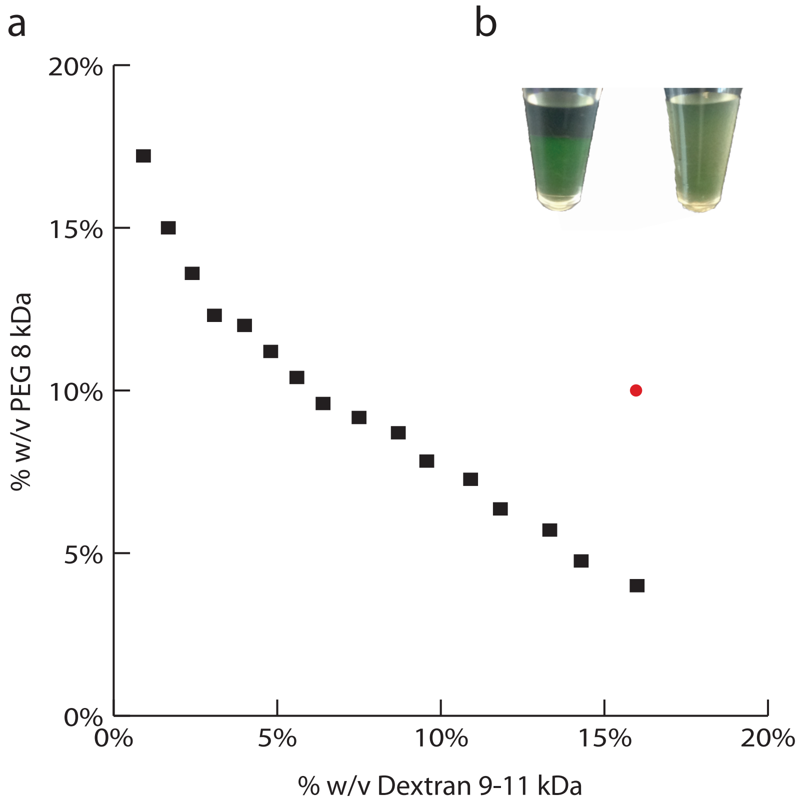

Fig. S1 (a) Experimentally derived phase diagram for a dextran 9-11 kDa/PEG 8 kDa ATPS sample at room temperature in 50 mM Tris-Cl pH 8 and 100 mM NaCl. The final system composition (red dot) was chosen such that the sample was in the two-phase regime. **(b)** 16% w/v dextran 9-11 kDa/10% w/v PEG 8 kDa ATPS samples with 5 μM 5′-6-FAM-labeled RNA 15-mer (5′-CCAGUCAGUCUACGC-3′) at room temperature in 50 mM Tris-Cl pH 8 and 100 mM NaCl. On the left, the system is clearly separated into two phases with the RNA in the bottom, dextran-rich phase. On the right, the system is composed of small, dispersed dextran-rich droplets in the bulk PEG-rich phase and PEG-rich droplets in the bulk dextran-rich phase and resulted in a turbid solution.

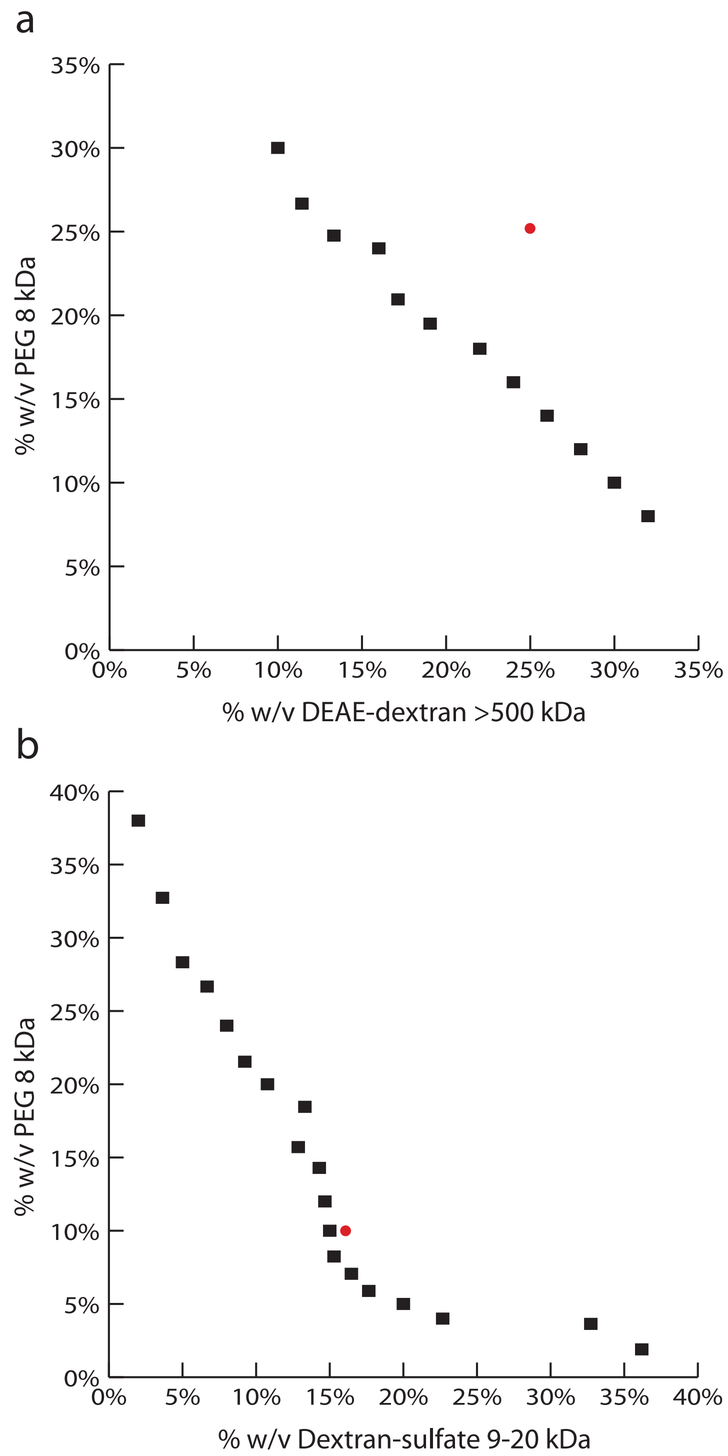

Fig. S2 (a) Experimentally derived phase diagram for a DEAE-dextran >500 kDa/PEG ATPS sample at room temperature in 100 mM Tris-Cl pH 8. **(b)** Experimentally derived phase diagram for a dextran-sulfate 9-20 kDa/PEG 8 kDa ATPS sample at room temperature in 50 mM Tris-Cl pH 8 and 100 mM NaCl. The red dots represent the final system compositions (25% w/v DEAE-Dextran >500 kDa/25% w/v PEG 8kDa and 16% w/v Dextran-Sulfate 9-20kDa/10% w/v PEG 8 kDa) used for all studies and were chosen such that the sample was in the two-phase regime of both systems, respectively.

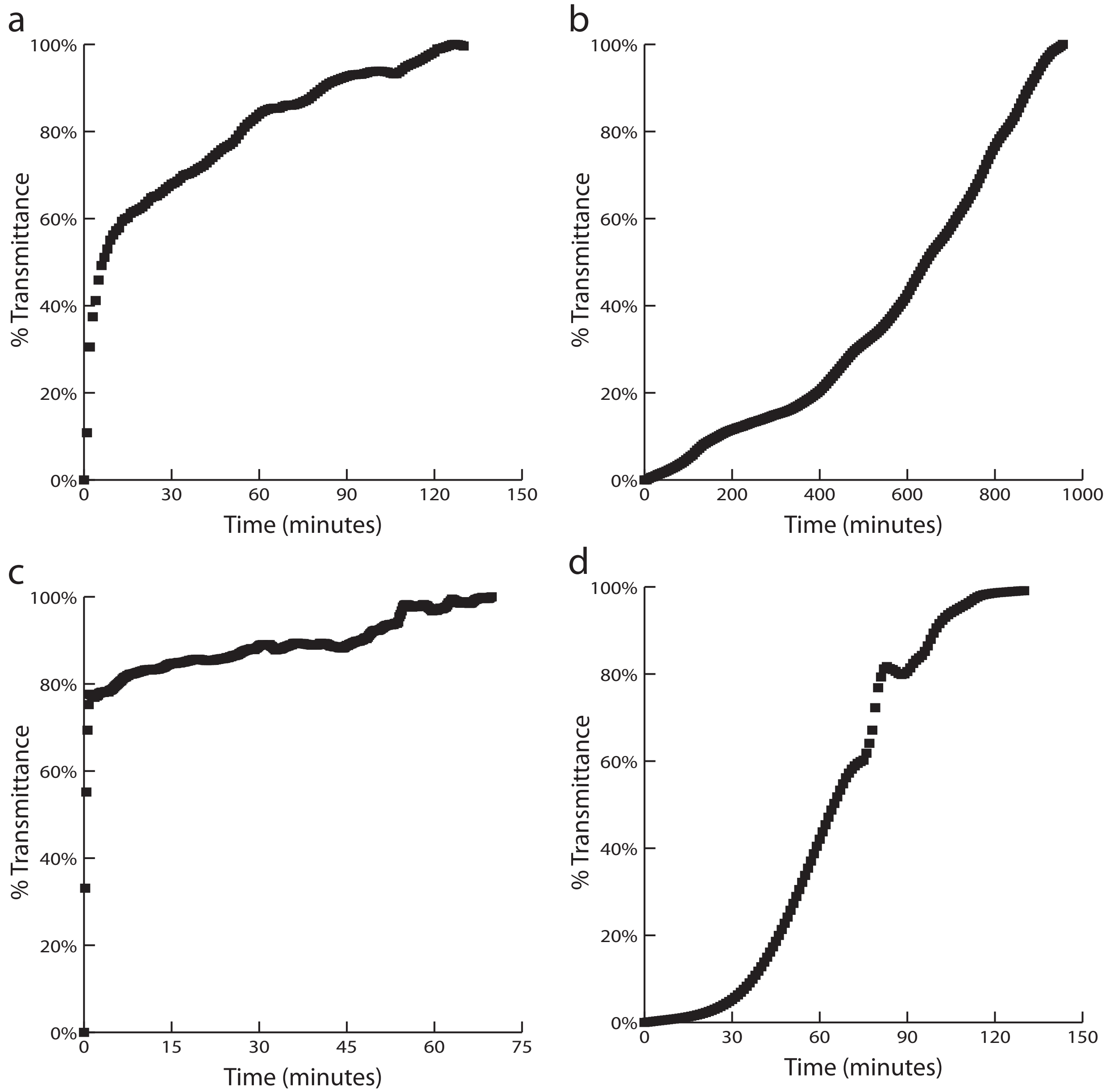

Fig. S3 Percent transmittance at room temperature over time of: **(a)** a 16% w/v dextran 9-11 kDa/10% w/v PEG 8 kDa ATPS sample at room temperature in 50 mM Tris-Cl pH 8 and 100 mM NaCl, **(b)** a 25% w/v DEAE-dextran >500 kDa/25% w/v PEG 8 kDa ATPS sample at room temperature in 100 mM Tris-Cl pH 8, **(c)** a 16% w/v dextran-sulfate 9-20 kDa/10% w/v PEG 8 kDa ATPS sample at room temperature in 50 mM Tris-Cl pH 8 and 100 mM NaCl, and **(d)** a 30 mM ATP/2% w/v pLys 4-15 kDa coacervate sample in 100 mM Tris-Cl pH 8 at room temperature. Average percent transmittance was determined for the 399 nm to 702 nm range and each sample was independently normalized against its own percent transmittance immediately after mixing (0%) and after both phases had completely separated (100%).

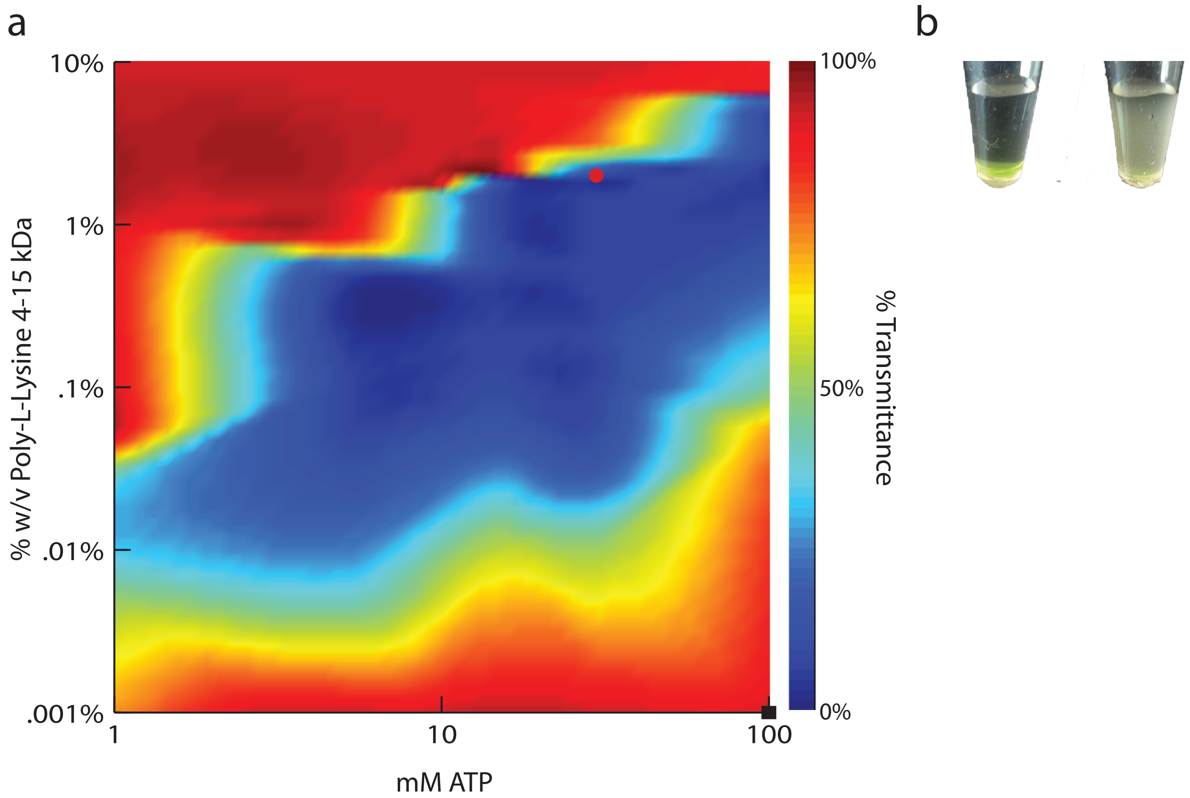

Fig. S4 (a) Experimentally derived and fitted percent transmittance profile for an ATP/pLys 4-15 kDa coacervate sample in 100 mM Tris-Cl pH 8. Note that there is a specific concentration range in which the coacervate will form. **(b)** 30 mM ATP/2% w/v pLys 4-15 kDa coacervate samples with 5 μM 5′-6-FAM-labeled RNA 15-mer (5′-CCAGUCAGUCUACGC-3′) at room temperature in 100 mM Tris-Cl pH 8. On the left, the system clearly separates into two phases with the RNA in the bottom, complex-enriched phase. On the right, the system is composed of small, dispersed complex-enriched droplets in bulk aqueous solution and resulted in a turbid solution.

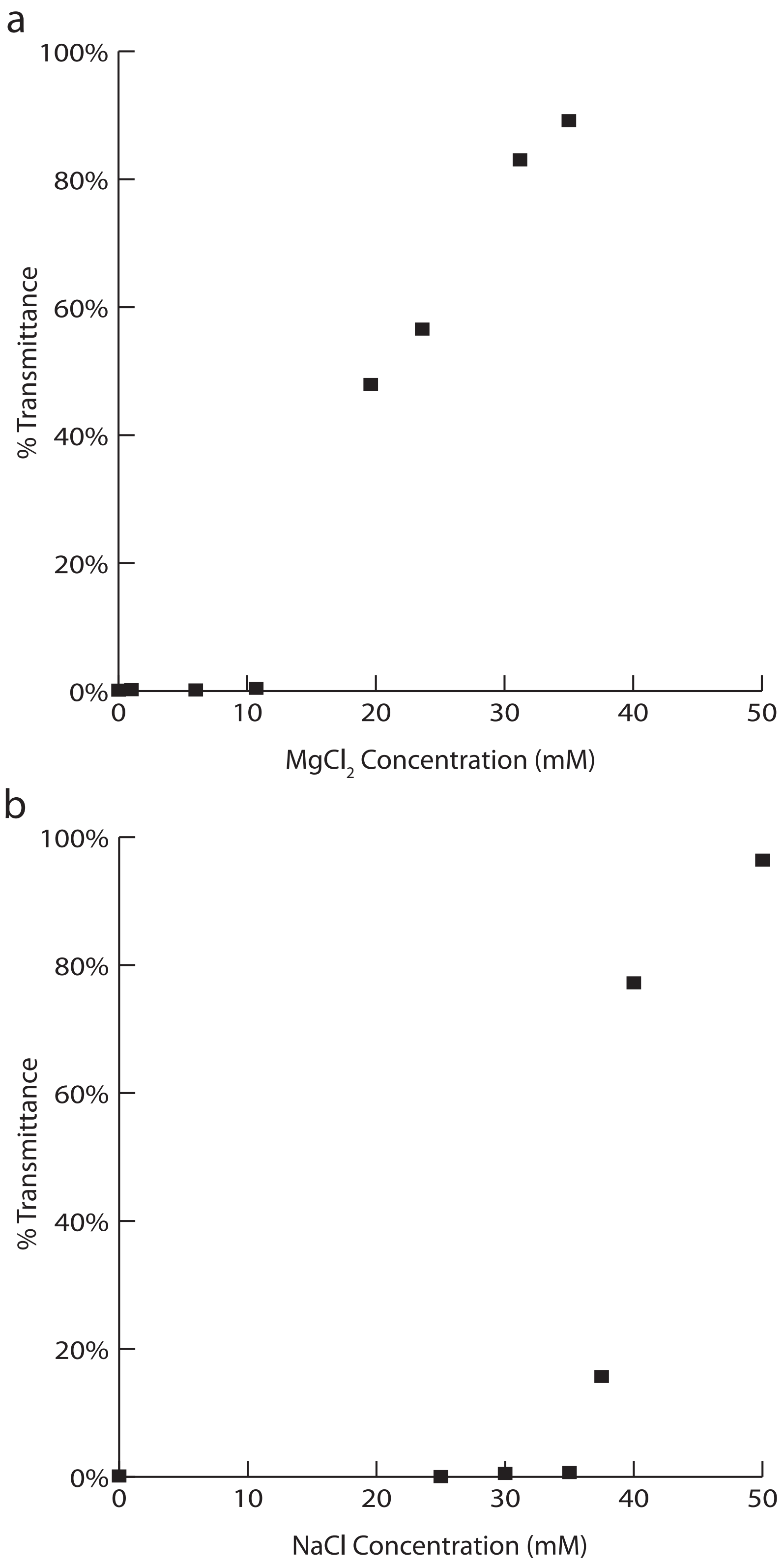

Fig. S5 Percent transmittance of a 30 mM ATP/2% w/v pLys 4-15 kDa coacervate sample in 100 mM Tris-Cl pH 8 at room temperature with increasing **(a)** $MgCl_2$ and **(b)** NaCl concentrations. At increasing salt concentrations, the charge interactions between the pLys and ATP are disrupted and the system starts to tend towards a one-phase system again.

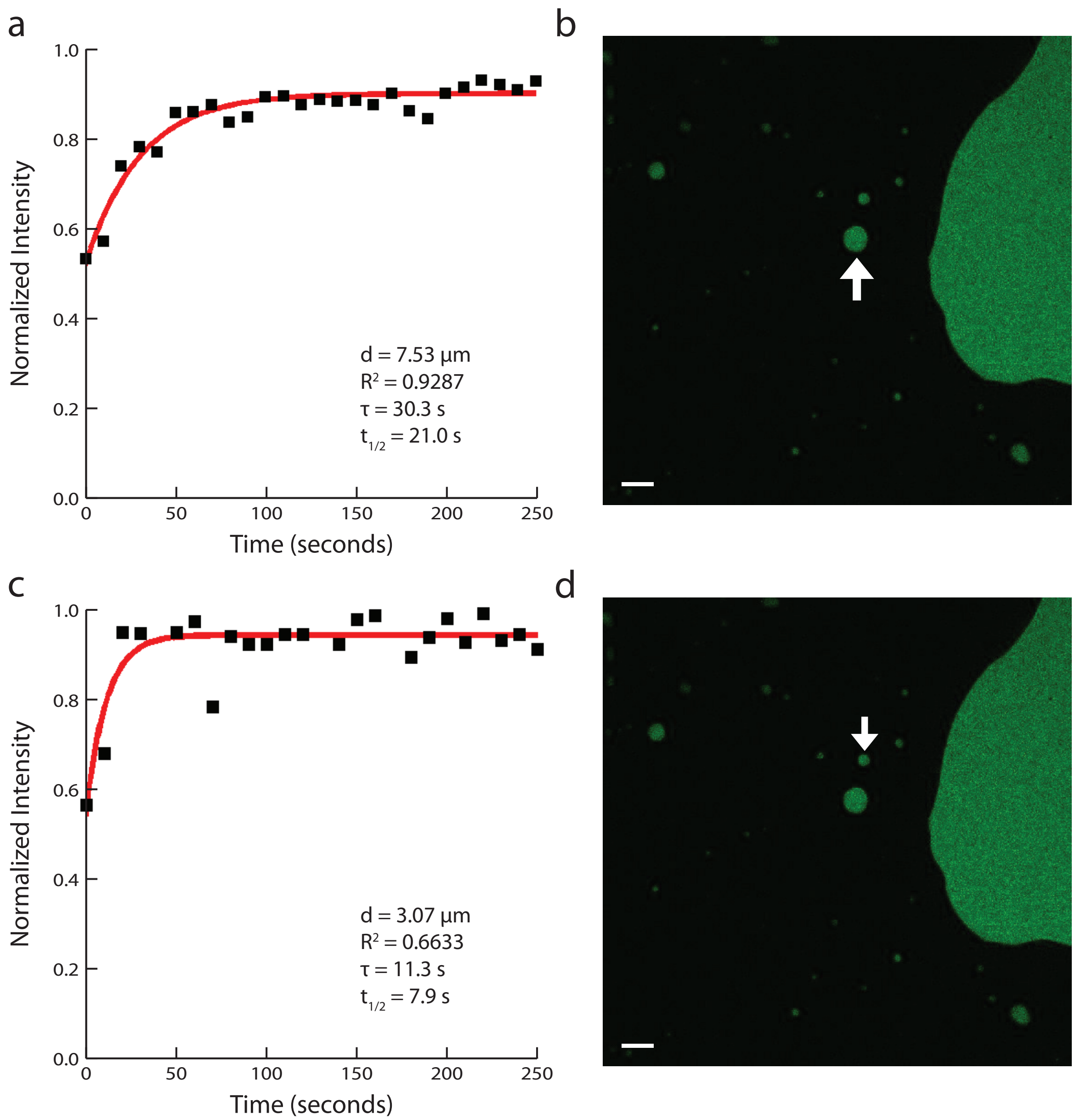

Fig. S6 Two FRAP curves and confocal microscope images of two different RNA-enriched droplets of a 30 mM ATP/2% w/v pLys 4-15 kDa coacervate sample with 5 μM 5′-6-FAM-labeled RNA 15-mer (5′-GCGUAGACUGACUGG-3′) at room temperature in 100 mM Tris-Cl pH 8. **(a)** Recovery curve and **(b)** confocal microscope image of 3.07 μm diameter droplet **(c)** Recovery curve and **(d)** confocal microscope image of 7.53 μm diameter droplet. See Table S3. Scale bars are each 10 μm.

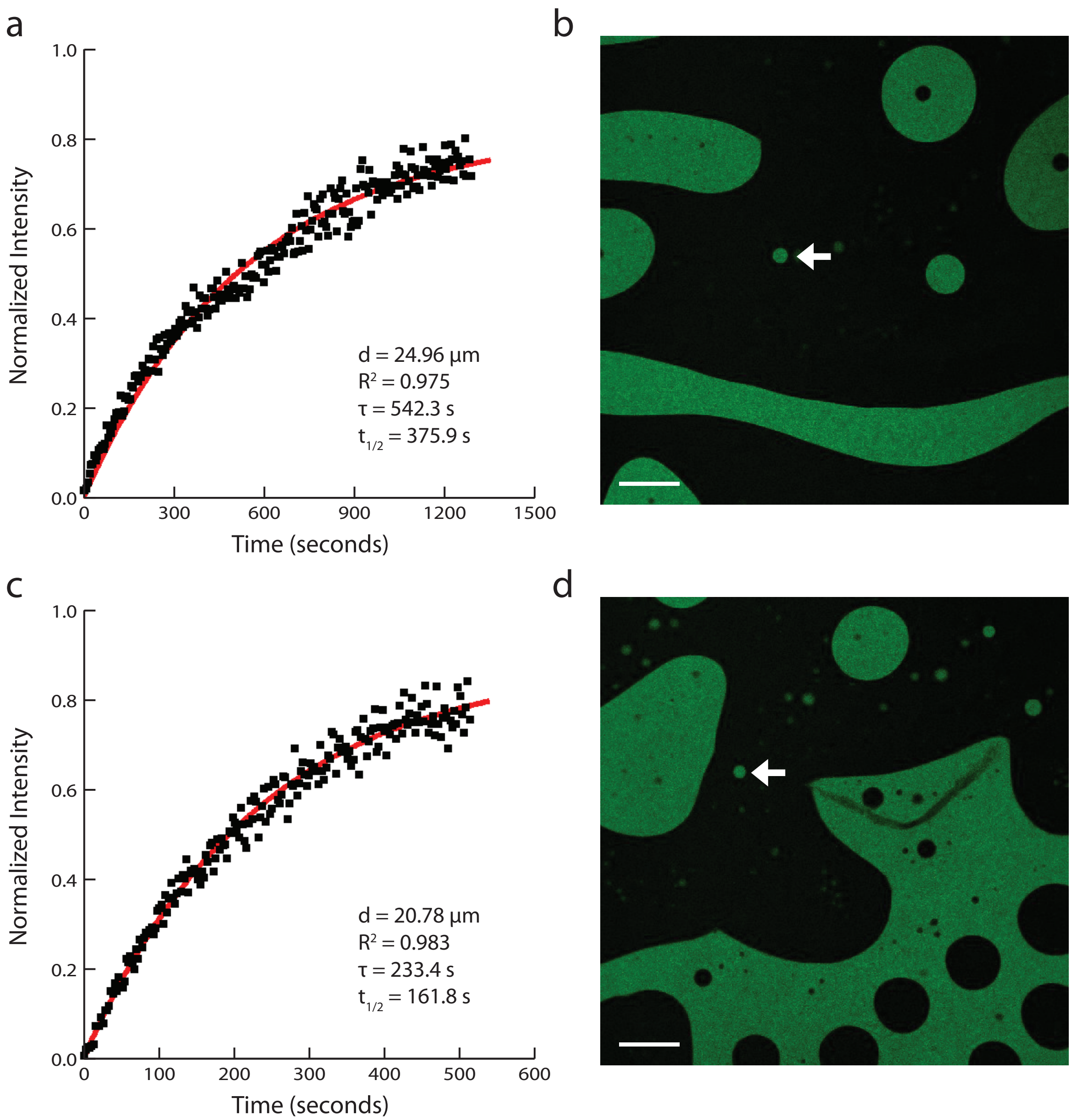

Fig. S7 Two FRAP curves and confocal microscopy images of two different RNA-enriched droplets of 16% w/v dextran 9-11 kDa/10% w/v PEG 8 kDa ATPS samples with 5 μM 5′-6-FAM-labeled RNA 15-mer (5′-GCGUAGACUGACUGG-3′) at room temperature in 50 mM Tris-Cl pH 8 and 100 mM NaCl. **(a)** Recovery curve and **(b)** confocal microscope image of 24.96 μm diameter droplet. **(c)** Recovery curve and **(d)** confocal microscope image of 20.78 μm diameter droplet. These two droplets were the largest sampled droplets in the dextran/PEG system (Table S3). Scale bars are each 100 μm.

Table S1

System	Degree of Partitioning	Standard Deviation
16 % Dextran 10kDa/10% PEG 8kDa	7.79	1.39
25% DEAE-Dextran 500kDa/25% PEG 8kDa	~100.0 (or greater)	~32.60
16% Dextran-Sulfate 9-20kDa/ 10% PEG 8kDa	23.71	3.58
30 mM ATP/2% Poly-L-Lysine 4-15 kDa	~141.0 (or greater)	21.38
16% Dextran 10kDa/10% PEG 8kDa (Non-labeled RNA)	9.53	0.25

Table S1 Degree of partitioning and the accompanying standard deviation (SD_{avg}) of 5 μM 5′-6-carboxyfluorescein (5′-6-FAM) labeled RNA 15-mer (5′-GCGUAGACUGACUGG-3′) into a specific phase in the various ATPSs and coacervate systems studied. The final row contained 5 μM the same non-labeled RNA 15-mer in order to confirm that the 6-FAM dye did not affect partitioning greatly. The degree of partitioning was calculated by dividing the absorbance at 495 nm of the RNA-enriched phase by the absorbance at 495 nm of the non-RNA-enriched phase. Absorbance was measured using a nanodrop spectrometer (*Methods*). Buffer conditions are identical to those mentioned in the captions for Fig. 1 and Fig. S1. The ATP/pLys system as well as the DEAE-dextran/PEG system exhibited such a high degree of partitioning that the RNA concentration in the non-complex-enriched phase and the PEG-rich phase, respectively, were below the limit of detection of the nanodrop spectrometer used.

Table S2

pLys Mol. Wt.	1-5 kDa	4-15 kDa	15-30 kDa
ATP	Y	Y	Y
ADP	N	Y	N
AMP	N	N	N
GTP	Y	Y	Y
GDP	Y	Y	Y
GMP	N	N	N
CTP	N	Y	Y
UTP	N	Y	Y
rATP	Y	Y	Y
rCTP	N	Y	Y
rUTP	N	Y	Y
rGTP	Y	Y	Y
dATP	N	Y	Y
dCTP	N	N	Y
dTTP	N	N	Y
dGTP	Y	Y	Y

Table S2 The ability for other alternatives to ATP (rows) and other molecular weights of pLys (columns) to form a coacervate. Blue indicates the successful ability for each pair to form a coacervate. Red indicates failure. Each different ATP alternative is at 30 mM and each different pLys molecular weight polymer is at 2% w/v. At other concentrations, more pairs than those indicated would also complex to form a coacervate.

Table S3

System	Diameter (μm)	A	τ (s)	C	R^2	$t_{1/2}$ (s)
16% Dextran 10kDa/10% PEG 8kDa	24.96	.8192	542.3	N/A	.9748	375.9
16% Dextran 10kDa/10% PEG 8kDa	20.78	.8828	233.4	N/A	.9829	161.8
16% Dextran 10kDa/10% PEG 8kDa	5.77	.4269	16.9	.5717	.6882	11.7
16% Dextran 10kDa/10% PEG 8kDa	5.77	.5001	20.4	.5599	.7681	14.2
16% Dextran 10kDa/10% PEG 8kDa	5.77	.4814	17.3	.5315	.7925	12.0
16% Dextran 10kDa/10% PEG 8kDa	5.77	.5115	15.4	.4772	.7683	10.7
16% Dextran 10kDa/10% PEG 8kDa	5.95	.4384	13.0	.4328	.7687	9.0
16% Dextran 10kDa/10% PEG 8kDa	5.95	.5436	16.7	.5556	.8188	11.5
16% Dextran 10kDa/10% PEG 8kDa	5.95	.4749	22.7	.5068	.8286	15.7
16% Dextran 10kDa/10% PEG 8kDa	5.95	.4704	11.6	.4342	.7391	8.1
25% DEAE-Dextran 500kDa/25% PEG 8kDa	8.65	.8553	27.7	N/A	.9136	19.2
25% DEAE-Dextran 500kDa/25% PEG 8kDa	9.48	.8476	27.2	N/A	.9135	18.8
16% Dextran-Sulfate 9-20kDa/10% PEG 8kDa	43.90	.8655	105.8	.1278	.9950	73.3
16% Dextran-Sulfate 9-20kDa/10% PEG 8kDa	34.99	.7827	77.6	.1749	.9730	53.8
30 mM ATP/2% Poly-L-Lysine 4-15 kDa	5.58	.5830	8.0	.4464	.8415	5.5
30 mM ATP/2% Poly-L-Lysine 4-15 kDa	5.58	.5864	7.1	.435	.8107	4.9
30 mM ATP/2% Poly-L-Lysine 4-15 kDa	5.58	.4026	7.1	.484	.7603	4.9
30 mM ATP/2% Poly-L-Lysine 4-15 kDa	9.21	.3976	17.6	.4798	.9342	12.2
30 mM ATP/2% Poly-L-Lysine 4-15 kDa	9.21	.4547	18.2	.4434	.9358	12.6
30 mM ATP/2% Poly-L-Lysine 4-15 kDa	9.21	.5223	13.8	.4194	.9059	9.5
30 mM ATP/2% Poly-L-Lysine 4-15 kDa	3.07	.4019	11.3	.5404	.6633	7.9
30 mM ATP/2% Poly-L-Lysine 4-15 kDa	3.07	.4202	12.2	.4884	.5803	8.4
30 mM ATP/2% Poly-L-Lysine 4-15 kDa	7.53	.3831	30.3	.5175	.9287	21.0
30 mM ATP/2% Poly-L-Lysine 4-15 kDa	7.53	.5208	19.6	.4208	.8665	13.6
30 mM ATP/2% Poly-L-Lysine 4-15 kDa	6.75	.5906	15.8	.2738	.9572	10.9
30 mM ATP/2% Poly-L-Lysine 4-15 kDa	6.75	.6824	16.2	.2767	.9429	11.2
30 mM ATP/2% Poly-L-Lysine 4-15 kDa	5.42	.6211	12.0	.3592	.9844	8.3
30 mM ATP/2% Poly-L-Lysine 4-15 kDa	5.42	.6413	17.0	.2702	.9036	11.8

Table S3 Fluorescence Recovery After Photobleaching (FRAP) recovery data for droplets containing 5 μM of a 5′-6-carboxyfluorescein (5′-6-FAM) labeled RNA 15-mer (5′-GCGUAGACUGACUGG-3′) sampled under the conditions indicated. Each curve (See Fig. 1, and SI Figs. 6 and 7 for representative recovery curves and confocal microscope images) was normalized to the intensities of a non-bleached droplet and the background within the same frame, to correct for nonspecific photobleaching during sampling, as well as to its initial intensity, to account for variability in the FRAP photobleaching step before the recovery step across runs (SI Note 1). Curves were fit to a single exponential recovery function ($y = A - A^*e^{x/\tau} + C$ where A and C are constant and τ is the recovery time constant). R^2 is the coefficient of determination. $t_{1/2}$ is the fluorescence recovery half-life.

Table S4

System	Diameter (μm)	A	τ (s)	C	R^2	$t_{1/2}$ (s)
16% Dextran 10kDa/10% PEG 8kDa	11.44	1.009	110.3	-.0085	.9966	76.4
16% Dextran 10kDa/10% PEG 8kDa	5.31	.9424	49.8	.0300	.9846	34.5
16% Dextran 10kDa/10% PEG 8kDa	5.31	.9471	50.9	.0094	.9846	35.3
16% Dextran 10kDa/10% PEG 8kDa	7.76	.9753	71.74	-.0389	.9897	49.7
16% Dextran 10kDa/10% PEG 8kDa	7.76	1.006	74.1	-.0483	.9915	51.3
30 mM ATP/2% Poly-L-Lysine 4-15 kDa	8.17	.6506	75.5	.3798	.8865	52.3
30 mM ATP/2% Poly-L-Lysine 4-15 kDa	5.93	.6859	37.8	.1935	.9347	26.2
30 mM ATP/2% Poly-L-Lysine 4-15 kDa	5.93	.7396	40.5	.2420	.9288	28.1
30 mM ATP/2% Poly-L-Lysine 4-15 kDa	5.93	.7077	35.2	.2368	.9268	24.4
30 mM ATP/2% Poly-L-Lysine 4-15 kDa	3.68	.6279	23.4	.2383	.9034	16.2
30 mM ATP/2% Poly-L-Lysine 4-15 kDa	3.68	.727	15.6	.2236	.8758	10.8

Table S4 Fluorescence Recovery After Photobleaching (FRAP) recovery data for droplets containing 5 μM of a 5′-6-carboxyfluorescein (5′-6-FAM) labeled RNA 50-mer (5′-CAUCUAGUUACCUCUAGGAUCUCAUGAUGCCUGAAGCGUAGACUGACUGG-3′) sampled under the conditions indicated. Each curve was normalized to the intensities of a non-bleached droplet and the background within the same frame, to correct for nonspecific photobleaching during sampling, as well as to its initial intensity, to account for variability in the FRAP photobleaching step before the recovery step across runs (SI Note 1). Curves were fit to a single exponential recovery function ($y = A - A*e^{x/\tau} + C$ where A and C are constant and τ is the recovery time constant). R^2 is the coefficient of determination. $t_{1/2}$ is the fluorescence recovery half-life.

Supplemental Movies

Please note that **Movies S1-S5** may be downloaded online from the journal website.

Movie S1. Representative Fluorescence Recovery After Photobleaching (FRAP) movie of a 16% w/v dextran 9-11 kDa/10% w/v PEG 8 kDa ATPS droplet with 5 μM 5'-6-FAM-labeled RNA 15-mer (5'-GCGUAGACUGACUGG-3') at room temperature in 50 mM Tris pH 8 and 100 mM NaCl (*Methods*). See Fig. 1a for a confocal microscope image and the recovery curve.

Movie S2. Representative Fluorescence Recovery After Photobleaching (FRAP) movie of a 25% w/v DEAE-dextran >500 kDa/25% w/v PEG 8 kDa ATPS droplet with 5 μM 5'-6-FAM-labeled RNA 15-mer (5'-GCGUAGACUGACUGG-3') at room temperature in 100 mM Tris pH 8 (*Methods*). See Fig. 1b for a confocal microscope image and the recovery curve.

Movie S3. Representative Fluorescence Recovery After Photobleaching (FRAP) movie of a 16% w/v dextran-sulfate 9-20 kDa/10% w/v PEG 8 kDa ATPS droplet with 5 μM 5'-6-FAM-labeled RNA 15-mer (5'-GCGUAGACUGACUGG-3') at room temperature in 50 mM Tris pH 8 and 100 mM NaCl at room temperature (*Methods*). See Fig. 1c for a confocal microscope image and the recovery curve.

Movie S4. Representative Fluorescence Recovery After Photobleaching (FRAP) movie of a 30 mM ATP/2% w/v pLys 4-15 kDa coacervate droplet with 5 μM 5'-6-FAM-labeled RNA 15-mer (5'-GCGUAGACUGACUGG-3') at room temperature in 100 mM Tris pH 8 (*Methods*). See Fig. 1d for a confocal microscope image and the recovery curve.

Movie S5. Representative Fluorescence Recovery After Photobleaching (FRAP) movie of a non-gel-filtered oleic acid vesicle produced in 200 mM Bicine-NaOH pH 8.5 containing 5'-6-FAM labeled RNA 15-mer (5'-CCAGUCAGUCUACGC-3') at room temperature (*Methods*). See Fig. 2 for the confocal microscope image before photobleaching and 10 minutes after photobleaching.
